# High-Resolution, High b-Value Computed Diffusion-Weighted Imaging Improves Detection of Pancreatic Ductal Adenocarcinoma

**DOI:** 10.3390/cancers14030470

**Published:** 2022-01-18

**Authors:** Felix N. Harder, Eva Jung, Sean McTavish, Anh Tu Van, Kilian Weiss, Sebastian Ziegelmayer, Joshua Gawlitza, Philip Gouder, Omar Kamal, Marcus R. Makowski, Fabian K. Lohöfer, Dimitrios C. Karampinos, Rickmer F. Braren

**Affiliations:** 1 Institute of Diagnostic and Interventional Radiology, School of Medicine, Technical University of Munich, 81675 Munich, Germany; Felix.Harder@tum.de (F.N.H.); ema.jung-moebus@gmx.de (E.J.); sean.mctavish@tum.de (S.M.); anh.van@tum.de (A.T.V.); s.ziegelmayer@tum.de (S.Z.); joshua.gawlitza@tum.de (J.G.); philip.gouder@tum.de (P.G.); o.kamal@aun.edu.eg (O.K.);marcus.makowski@tum.de (M.R.M.); fabian.lohoefer@tum.de (F.K.L.);dimitrios.karampinos@tum.de (D.C.K.); 2 Philips GmbH, Röntgenstrasse 22, 22335 Hamburg, Germany; kilian.weiss@philips.com

**Keywords:** computed diffusion-weighted imaging, high-resolution diffusion-weighted imaging, pancreas, pancreatic ductal adenocarcinoma, magnetic resonance imaging

## Abstract

**Simple Summary:**

High b-value diffusion-weighted imaging (DWI) has shown benefits in the diagnostic workup of pancreatic ductal adenocarcinoma (PDAC). However, technical and practical limitations restrict its widespread application in clinical routine. To overcome these limitations, computed high b-value DWI (cDWI) has been proposed, although there is a potential drawback of lower image quality. Recently, high-resolution (i.e., reduced field-of-view, rFOV) DWI has been proposed to ameliorate image quality and lesion detection in PDAC. We investigated the potential of combining high-resolution and computed high b-value DWI (r-cDWI) for the visualization of PDAC at a b-value of 1000 s/mm^2^. We found that the r-cDWI1000 outperformed both conventional computed (i.e., full field-of-view, fFOV) and acquired high-resolution DWI (f-aDWI1000 and r-aDWI1000) in the visualization of PDAC. Our results indicate the potential clinical benefits of high-resolution, high b-value computed DWI in PDAC imaging through enhanced lesion detection and reduced acquisition time.

**Abstract:**

Background: Our purpose was to investigate the potential of high-resolution, high b-value computed DWI (cDWI) in pancreatic ductal adenocarcinoma (PDAC) detection. Materials and Methods: We retrospectively enrolled 44 patients with confirmed PDAC. Respiratory-triggered, diffusion-weighted, single-shot echo-planar imaging (ss-EPI) with both conventional (i.e., full field-of-view, 3 × 3 × 4 mm voxel size, b = 0, 50, 300, 600 s/mm^2^) and high-resolution (i.e., reduced field-of-view, 2.5 × 2.5 × 3 mm voxel size, b = 0, 50, 300, 600, 1000 s/mm^2^) imaging was performed for suspected PDAC. cDWI datasets at b = 1000 s/mm^2^ were generated for the conventional and high-resolution datasets. Three radiologists were asked to subjectively rate (on a Likert scale of 1–4) the following metrics: image quality, lesion detection and delineation, and lesion-to-pancreas intensity relation. Furthermore, the following quantitative image parameters were assessed: apparent signal-to-noise ratio (aSNR), contrast-to-noise ratio (aCNR), and lesion-to-pancreas contrast ratio (CR). Results: High-resolution, high b-value computed DWI (r-cDWI1000) enabled significant improvement in lesion detection and a higher incidence of a high lesion-to-pancreas intensity relation (type 1, clear hyperintense) compared to conventional high b-value computed and high-resolution high b-value acquired DWI (f-cDWI1000 and r-aDWI1000, respectively). Image quality was rated inferior in the r-cDWI1000 datasets compared to r-aDWI1000. Furthermore, the aCNR and CR were higher in the r-cDWI1000 datasets than in f-cDWI1000 and r-aDWI1000. Conclusion: High-resolution, high b-value computed DWI provides significantly better visualization of PDAC compared to the conventional high b-value computed and high-resolution high b-value images acquired by DWI.

## 1. Introduction

Pancreatic ductal adenocarcinoma (PDAC) is characterized by a grim prognosis, reflected by a five-year survival rate of 9% [1]. By 2030, PDAC will be the second leading cause of cancer-related deaths in the USA [2]. However, prolonged survival rates have been documented in patients with early detected small tumors [3]. Magnetic resonance imaging (MRI) renders radiation-free, non-invasive tumor detection with high sensitivity and specificity and is of particular value for small tumors of less than 2 cm [4,5]. Within multiparametric MRI (mpMRI), diffusion weighted imaging (DWI) is widely accepted as a key technique in oncologic imaging for a variety of tumor entities [6]. In brief, DWI is a contrast-agent-free MRI technique, able to detect the microscopic random Brownian motion of water protons [7]. Impeded diffusion occurs in a variety of pathologic conditions, such as inflammatory, fibrotic, or neoplastic processes. Previous studies have emphasized the diagnostic value of DWI in PDAC imaging [8,9]. However, the exact delineation of tumor margins can be obscured by surrounding pancreatitis with consecutive hyperintense parenchyma [10]. Furthermore, endoscopic-ultrasound-based tumor sampling and histopathological proof are still required in most cases of primary diagnosis, limiting the impact of non-invasive methods such as cDWI for the detection of suspected lesions and exact staging prior to therapeutic intervention [11]. Previous studies have reported improved pancreatic tumor detection utilizing high b-value DWI [12,13]. High b-value DWI reduces the T2 shine-through effect (i.e., incomplete suppression of the T2-signal) and enables higher contrast between the tumor and the surrounding pancreatic parenchyma, leading to better tumor detection. However, high b-value DWI is subject to a variety of restrictions in clinical routine, limiting its widespread application. In particular, longer acquisition times not only lead to patient discomfort, but also compromise the image quality due to motion artifacts and decreased signal-to-noise ratio (SNR) [14].

Artificially generated computed high b-values from at least two acquired lower b-values based on a voxel-wise mono-exponential fit have been proposed as a means to overcome these limitations. Hence, cDWI combines the previously mentioned advantages of high b-value DWI with reduced image acquisition time. 

Previous studies have reported increased tumor detection in cDWI images for prostate, pancreatic, and hepatic cancer [13,15,16]. However, contradictory findings were reported by Tamura et al. in a study on cDWI in breast cancer [17]. Particularly, degraded image quality remains a major limitation in cDWI [12,13,17,18].

High-resolution DWI (i.e., reduced field-of-view DWI (rDWI)) has been introduced to significantly improve image quality and diagnostic certainty in pancreatic pathologies, due to reduced motion and susceptibility artifacts, as well as a more precise method of lesion detection [19,20]. Recent studies highlighted the potential of rDWI and cDWI in breast cancer and prostate cancer [21,22]. Yet, no previous study has investigated the potential benefits of combining rDWI and cDWI in pancreatic cancer imaging. 

Aiming to combine both the advantages of rDWI and cDWI in pancreatic cancer imaging, we hypothesized improved image quality and tumor detection in high-resolution high b-value computed DWI (r-cDWI).

## 2. Materials and Methods

### 2.1. Patient Cohort and Study Design

This was a retrospective, single-center study. All patients were referred to the surgical or gastroenterological unit of our tertiary hospital for suspected pancreatic cancer. Patients who underwent MRI examination were included. The study was conducted in accordance with the Declaration of Helsinki. Approval was granted by the local ethics committee (245/19 S-SR). The requirement for informed consent was waived.

The following clinical data were obtained for every patient: age at diagnosis, sex, tumor site (pancreatic head, body, or tail), and initial tumor markers for CEA and CA 19-9.

### 2.2. Data Acquisition and Postprocessing

MR imaging was performed on a 3T scanner (Philips Ingenia Elition; Philips Medical Systems, Best, The Netherlands) using a combination of a 16-channel torso coil array and an inbuilt table posterior 12-channel coil array.

The following sequences were performed: (1) T2-weighted (T2w) turbo spin echo (TSE); (2) 2D diffusion-weighted (DW), single-shot echo planar imaging (ss-EPI) with conventional (i.e., full field-of-view, fFOV) covering of the upper abdomen; and (3) a 2D ss-EPI sequence with a high-resolution (i.e., reduced field-of-view, rFOV) covering of the pancreas. For both DWI datasets, the following b-values (averages) were acquired: 0 (1), 50 (1), 300 (2), and 600 (5) s/mm^2^. Furthermore, an additional high b-value at b = 1000 (11) s/mm^2^ was acquired in the rFOV datasets (r-aDWI1000). All images were acquired in axial planes. The fFOV datasets were acquired with phase-encoding A/P and an in-plane FOV of 420 × 370 mm (RL/AP) at a resolution of 3 × 3 × 4 mm and were reconstructed to a matrix of 140 × 121 pixels. 

Further imaging parameters for the fFOV images were as follows: TR = 1850 ms, TE = 72 ms, SENSE R = 2.5 (A/P), no partial Fourier, bandwidth = 2304 Hz/pixel, 43 slices acquired in 3 packages, and scan time = 4:30 min for a respiratory cycle with a period of 3 s.

Reducing the field-of-view aimed to increase spatial resolution without increasing off-resonance-induced geometric distortions by increasing the bandwidth per pixel in the phase-encoding direction. The rFOV imaging was acquired with phase encoding in the L/R direction. Exploiting the higher number of receiver coils in the L/R direction compared to the A/P direction enables a higher parallel-imaging acceleration factor; thus, the rFOV can be employed without aliasing. Image unfolding was performed using regularized SENSE. The g-factor noise enhancement was reduced by incorporating prior knowledge of the expected signal levels. Further rFOV imaging parameters included: FOV of 300 × 300 mm (AP/LR) at a resolution of 2.5 × 2.5 × 3 mm, TR = 1627 ms, TE = 67 ms, SENSE R = 2.5 (L/R, over the prescribed reduced FOV), no partial Fourier, bandwidth = 2817 Hz/pixel, 20 slices acquired in 2 packages, scan time 3:18 min (b0, 600 s/mm^2^), and 15:00 min (b0, 1000 s/mm^2^) for a respiratory cycle with a period of 3 s. 

The cDWI datasets were generated by applying a dedicated software tool (Philips IntelliSpace Portal, Philips Medical Systems, Best, The Netherlands). Then, b-values of 0, 50, 300, and 600 s/mm^2^ were used to calculate the cDWI datasets at a b-value of 1000 s/mm^2^ based on a mono-exponential fit model for both the fFOV (f-cDWI1000) and rFOV (r-cDWI1000) datasets. 

### 2.3. Image Analysis 

To assess performances, (1) conventional and high-resolution, high b-value computed datasets (f-cDWI1000 and r-cDWI1000) were compared to the corresponding standard b-value acquired datasets (f-aDWI600 and r-aDWI600); (2) to each other; and (3) against high-resolution, high b-value acquired datasets (r-aDWI1000).

### 2.4. Qualitative Analysis 

The qualitative image analysis was performed by three readers with four (reader 1, reader 2) and two (reader 3) years of experience. The readers were blinded to each other’s results. The diagnostic quality was verified for all images by reader 1 prior to the readings. The readers were allowed to zoom in, adjust window levels, and use T2w images for anatomic orientation. 

The qualitative analysis was performed based on a 4-point Likert scale. The following parameters were assessed: overall image quality (4 = excellent, 3 = good, 2 = fair, and 1 = poor), lesion detection and delineation (4 = excellent, 3 = good, 2 = fair, and 1 = poor), and DWI signal intensity (SI) type of the PDAC (Type 1: clear hyperintensity to normal parenchyma; Type 2: hyperintense, but distal border obscure because of hyperintense parenchyma due to pancreatitis, Type 3: isointense, and Type 4: hypointense), according to Fukukura et al. [23]. The ratings from reader 1 are displayed in the Results section.

### 2.5. Quantitative Analysis 

Regions of interest (ROIs) measuring 5 mm were manually placed in the tumor and in the healthy-appearing pancreatic tissue next to the tumor in the pancreatic head and tail. ROIs were placed in the b600 s/mm^2^ datasets and then copied and pasted to the corresponding computed datasets, as well as the directly acquired rFOV-DWI datasets at b = 1000 s/mm^2^. Manual correction was performed when necessary. The mean signal intensity (SI) and standard deviation (SD) inside the ROIs were recorded. 

The apparent signal-to-noise ratio (aSNR) was calculated as follows:

aSNR = SI_normal parenchyma_/SD_normal parenchyma_.

The apparent contrast-to-noise ratio (aCNR) was calculated as follows:

aCNR = (SI_tumor_ − SI_normal parenchyma_)/SD_normal parenchyma_.

The contrast ratio between the tumor and the adjacent normal parenchyma was calculated as follows:

CR = SI_lesion_/SI_normal pancreas_

### 2.6. Statistical Analysis

The Kolmogorov–Smirnov test was applied to test for normal distribution. The qualitative metrics were analyzed pairwise using the Wilcoxon-signed-rank test. The Friedman test was performed to analyze the distribution of DWI subtypes between datasets and the Dunn–Bonferroni test was used for multiple comparison. Inter-rater agreement was calculated using Fleiss’ kappa and considered as slight: κ = 0.00–0.20, fair: κ = 0.21–0.40, moderate: κ = 0.41–0.60, substantial for κ = 0.61–0.80, and almost perfect: κ = 0.81–1.00. The *p*-values ≤ 0.05 were considered statistically significant. All statistics were performed in IBM SPSS (IBM Corp., Armonk, NY, USA), version 25.

## 3. Results

### 3.1. Patient Characteristics

**Figure A1:**
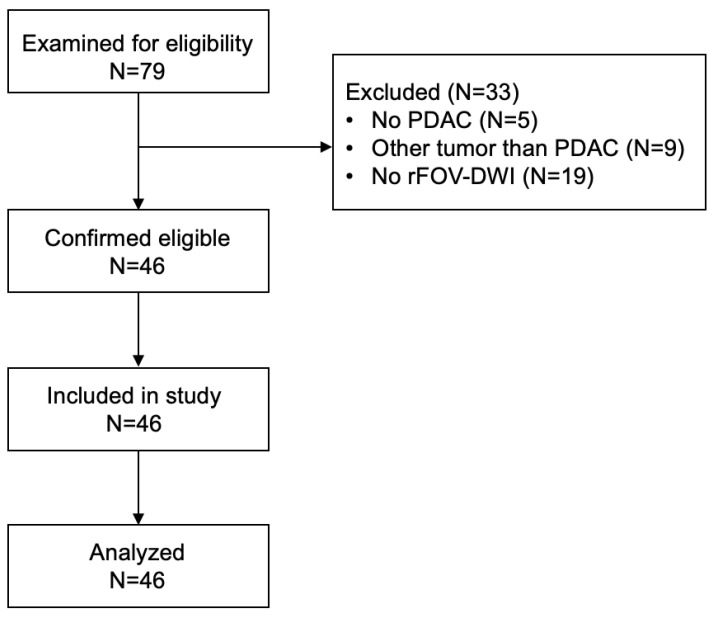
Patient inclusion flowchart.

Seventy-nine patients with suspected pancreatic cancer were examined for eligibility. Thirty-three patients were excluded for the following reasons: no PDAC in the histopathological examination (*n* = 5), a tumor other than PDAC (*n* = 9), or no acquired high-resolution DWI (*n* = 19). Finally, a total of 46 patients (median age: 68 years ± 11, 19 women, and 27 men) were enrolled in this study. The patient inclusion flow chart can be found in Figure A1 (Appendix A). Table 1 displays detailed patient characteristics. In 31 patients, the diagnosis was confirmed histopathologically after resection or fine needle aspiration (FNA). In the remaining 15 patients, other imaging modalities (i.e., CT, ERCP, or endoscopic ultrasound) in synopsis with the clinical course, laboratory parameters, and follow-up imaging confirmed the diagnosis. All images were acquired between March 2018 and April 2021. 

### 3.2. Qualitative Parameters

#### 3.2.1. Image Quality

**Figure 1:**
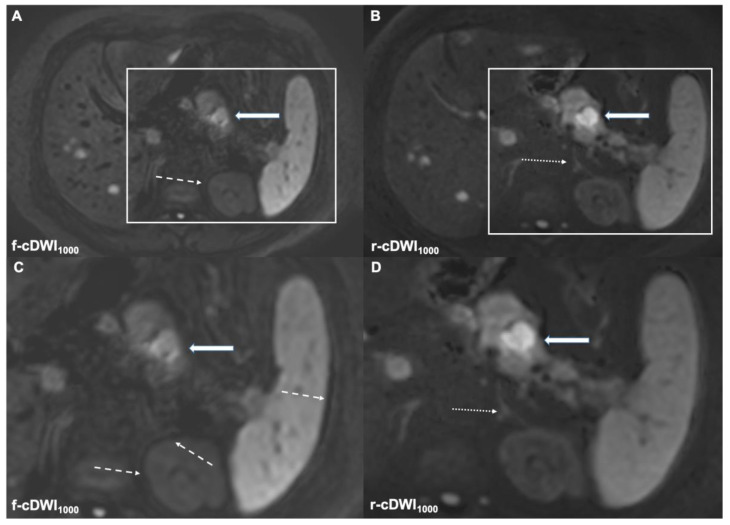
The high-resolution rFOV-DWI (**B**,**D**) yields better overall image quality compared to the conventional fFOV-DWI (**A**,**C**). Reduced distortion and motion artifacts enable better tumor delineation in the r-cDWI1000 (bold arrow in (**B**,**D**). Increased misregistration artifacts are depicted in the f-cDWI1000 images (dotted arrows in (**A**,**C**)). Additionally, note the better delineation of the left adrenal gland in the r-cDWI1000 images (dotted arrow in (**B**,**D**)).

The image quality was significantly higher in the acquired conventional and high-resolution standard b-value datasets than in the respective computed high b-value datasets (f-aDWI600 2.98 ± 0.61, f-cDWI1000 2.15 ± 0.69, and *p* < 0.00001; r-aDWI600 3.52 ± 0.62, r-cDWI1000 3.26 ± 0.6, and *p* = 0.00222). Furthermore, high-resolution, high b-value DWI outperformed the conventional high b-value computed datasets (r-cDWI1000 3.26 ± 0.6, f-2.15 ± 0.69, and *p* < 0.00001) (Figure 1). Finally, image quality was significantly higher in the acquired conventional datasets than in the computed high-resolution high b-value datasets (r-aDWI1000 3.5 ± 0.58, r-cDWI1000 3.26 ± 0.6, and *p* = 0.0096) (Table 2). Fleiss’ kappa yielded an inter-rater agreement of almost perfect (κ = 0.84–0.89).

#### 3.2.2. Lesion Detection

**Figure 2:**
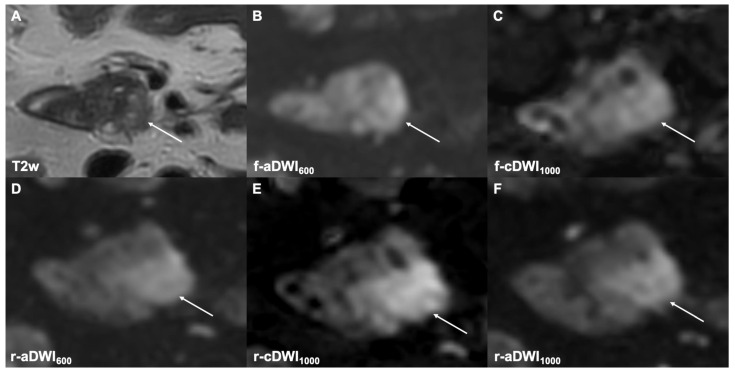
The axial T2w image shows a small tumor in the pancreatic head (**A**, white arrow) with an unclear border (type 2) to the adjacent parenchyma in both the f-aDWI600 (**B**) and f-cDWI1000 (**C**) images. Better lesion detection and tumor-to-parenchyma contrast (type 1) are depicted in the r-cDWI1000 (**E**) image compared to the r-aDWI600 (**D**) image and the directly acquired image at b = 1000 s/mm^2^ (**F**). The labeling in image (**E**) has been corrected.

**Figure 3:**
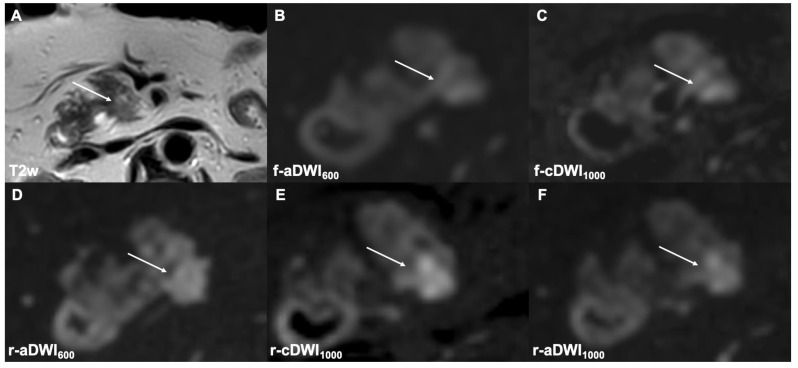
The axial T2w image (**A**) depicts a small pancreatic tumor in the uncinate process. The tumor is only poorly detected in the f-aDWI600 (**B**), f-cDWI1000 (**C**), and r-aDWI600 (**D**) images. The r-cDWI1000 (**E**) image yields superior tumor delineation in comparison to directly acquired images at b = 1000 s/mm^2^ r-aDWI1000 (**F**). The labeling in image (**E**) has been corrected.

**Figure 4:**
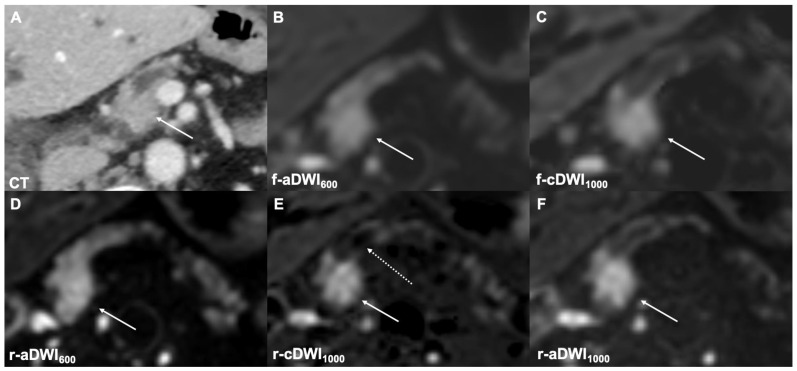
A CT image of a tumor in the pancreatic head causing obstruction of the main pancreatic duct (**A**). No clear tumor detection (type 3) is visible in the acquired images at b = 600 s/mm^2^ (**B**,**D**). The tumor conspicuity is superior in the images at b = 1000 s/mm^2^ (**C**,**E**,**F**). The fluid signal in the main pancreatic duct is best-suppressed in the r-cDWI1000 images ((**E**), dotted arrow). The labeling in image (**E**) has been corrected.

In contrast, significantly improved lesion detection was obtained in computed conventional and high-resolution high b-value datasets than in the respective acquired standard b-value datasets (f-aDWI600 2.34 ± 0.93, f-cDWI1000 2.78 ± 0.88, *p* = 0.00022; r-aDWI600 2.7 ± 0.91, r-cDWI1000 3.67 ± 0.7, *p* < 0.00001). Furthermore, computed high-resolution datasets outperformed conventional datasets at high b-values (r-cDWI1000 3.67 ± 0.7, f-cDWI1000 2.78 ± 0.88, *p* < 0.00001). Finally, computed high-resolution, high b-value outperformed the respective acquired datasets in lesion detection (r-cDWI1000 3.67 ± 0.7, r-aDWI1000 3.47 ± 0.68, *p* = 0.041) (Figure 2, Figure 3 and Figure 4). Fleiss’ kappa revealed high inter-rater agreement (κ = 0.86–0.94).

#### 3.2.3. DWI Type

A higher incidence of type 1 tumors was found in the computed conventional and high-resolution, high b-value datasets than in the respective acquired standard b-value datasets (Table 3). However, direct comparison revealed significance only for the high-resolution datasets (f-aDWI600 vs. f-cDWI1000 and *p* = 0.277; r-aDWI600 vs. r-cDWI1000 and *p* = 0.008). Furthermore, a significantly higher incidence of type 1 tumors was detected in the computed high-resolution datasets than in the conventional high b-value datasets (*p* = 0.006). Again, a higher, yet not significant, incidence of type 1 tumors was found in the computed datasets than in the acquired high-resolution high b-value datasets (*p* = 0.41). Fleiss’ kappa revealed a high inter-rater agreement (κ = 0.92–0.96).

### 3.3. Quantitative Parameters

#### 3.3.1. aSNR

The aSNR was significantly higher in the acquired standard b-value dataset than in the computed high b-value for both the high-resolution and conventional datasets (f-aDWI600 17.1 ± 7.15, f-cDWI1000 15.38 ± 8.75, and *p* = 0.01778; r-aDWI600 14.28 ± 5.69, r-cDWI1000 11.29 ± 4.06, and *p* = 0.00168). Additionally, the aSNR was significantly higher in f-cDWI1000 than in r-cDWI1000 (15.38 ± 8.75, 11.29 ± 4.06, and *p* = 0.00804). The r-cDWI1000 outperformed the r-aDWI1000 (11.29 ± 4.06, 9.33 ± 2.54, and *p* = 0.00438). (Table 4).

#### 3.3.2. aCNR

The aCNR was significantly higher in the computed high b-value dataset than in the acquired standard b-value datasets for both the high-resolution and conventional datasets (f-aDWI600 7.97 ± 5.77, f-cDWI1000 11.56 ± 9.9, and *p* = 0.0035; r-aDWI600 9.92 ± 8.21, r-cDWI1000 14.97 ± 9.16, and *p* = 0.00138). 

Furthermore, the aCNR was significantly higher in r-cDWI1000 than in f-cDWI1000 (14.97 ± 9.16, 9.92 ± 8.21, and *p* = 0.02088). r-cDWI1000 outperformed r-aDWI1000 (14.97 ± 9.16, 8.99 ± 5.89, and *p* < 0.00001). (Table 4).

#### 3.3.3. CR

The CR between the tumor and the proximal, as well as the distal pancreatic parenchyma, was significantly higher in the high b-value dataset than in the acquired standard b-value datasets for both the high-resolution and conventional datasets (proximal: f-aDWI600 1.51 ± 0.45, f-cDWI1000 1.79 ± 0.64, and *p* = 0.00028; r-aDWI600 1.8 ± 0.64, rDWI1000 2.48 ± 1.02, and *p* < 0.00001; distal: f-aDWI600 1.56 ± 0.49, f-cDWI1000 2.05 ± 1.18, and *p* = 0.00014; r-aDWI600 1.76 ± 0.66, r-cDWI1000 2.68 ± 1.32, and *p* < 0.00001). Comparing the f-cDWI1000 and r-cDWI1000 images revealed a significantly higher CR in the rFOV datasets (proximal: <0.00001 and distal: <0.00001). r-cDWI1000 outperformed r-aDWI1000 (proximal: r-cDWI1000 2.48 ± 1.02, r-aDWI1000 2.04 ± 0.81 and *p* = 0.00128; distal: r-cDWI1000 2.68 ± 1.32, r-aDWI1000 2.08 ± 0.74, and *p* = 0.0002). (Table 4).

## 4. Discussion

In this study, we assessed qualitative and quantitative image parameters in computed DWI (cDWI) at a high b-value derived from conventional and high-resolution DWI datasets. The computed high-resolution, high b-value DWI (r-cDWI1000) significantly outperformed the computed conventional high b-value DWI (f-cDWI1000) with regard to image quality, lesion discernibility, CNR, and CR. Furthermore, r-cDWI1000 outperformed the directly acquired high-resolution, high b-value DWI (r-aDWI1000).

In our study, lesion detection was significantly improved in the cDWI datasets compared to the acquired datasets with the standard b-value, holding true for both conventional (i.e., full field-of-view DWI (f-cDWI)) and high-resolution (reduced field-of-view DWI (r-cDWI)) datasets. Notably, in direct comparison, r-cDWI1000 significantly outperformed f-cDWI1000 in lesion detection. Better lesion detection at higher b-values can be explained by a reduced T2 shine-through effect of the surrounding benign pancreatic parenchyma relating to the tumor. Furthermore, tumor conspicuity on the cDWI was shown to improve with increasing quality of the source images, which is reflected by the higher resolution in high-resolution DWI [17].

The subjectively rated higher lesion detection is supported by a significant increase in lesion CR and CNR found in the r-cDWI1000 datasets compared to the f-cDWI1000 datasets in our study. Additionally, the incidence of clear hyperintense PDAC was significantly higher in the r-cDWI1000 datasets, confirming this finding. Our results are in line with those of a previous study on the value of cDWI in PDAC by Fukukura et al. [12]. Further results from two recent studies point in the same direction; yet, in these studies, b-values of the acquired and computed DWI were not the same, thus limiting a side-by-side comparison [13,24]. 

Previous studies have reported lower image quality with cDWI than in acquired DWI (aDWI) images [12,13,18,24]. Similarly, image quality was significantly degraded in the cDWI datasets compared to the conventional datasets at standard b-value in our study. We attributed this finding to misregistration artifacts resulting from misalignment between the low-b-value images due to bulk motion [25]. Advanced image registration tools can provide help to overcome this issue [15]. Previous studies on cDWI of the prostate reported equal or better image quality [16,26,27]. Yet, the high susceptibility of the pancreas to breathing artifacts and bowel motion might explain the divergence between results. Nevertheless, direct comparison revealed significantly better image quality for the r-cDWI1000 compared to the f-cDWI1000. This finding can be explained by the reduced susceptibility to motion artifacts, distortion, and partial volume, which has been reported for high-resolution images in direct comparison with conventional DWI [19,28,29]. Our results indicate that the combination of high-resolution and computed DWI may significantly improve image quality.

Both conventional and high-resolution cDWI datasets revealed significantly lower aSNR compared to the aDWI datasets at the standard b-value. Tamamura et al. also reported lower SNR for cDWI images compared to aDWI images in a study on breast cancer [17]. Gatidis et al. found background SI variance in cDWI images increased monotonically with increasing b-values [18]. Additionally, computed b-values cannot be increased indefinitely. Setting the b-value too high will result in a decrease in the SNR [25]. Hence, further prospective studies should evaluate the optimal gap between the maximum acquired b-value and the b-value set for the calculated cDWI images. 

In our study, the aSNR in the r-cDWI1000 datasets was significantly lower than in the f-cDWI1000 datasets. 

This can be explained by the higher plane resolution in the high-resolution sequence. The SNR increases linearly with voxel size. The high-resolution sequence with its inherently smaller voxel size is consequently more prone to a reduced SNR, and a lower SNR in comparison has been reported previously [19,30,31]. 

Intrinsically low SNR is a major shortcoming in high b-value DWI owing to the application of large diffusion-sensitizing gradients [32]. This might serve as an explanation for the significantly higher aSNR in the r-cDWI1000 datasets than in the r-aDWI1000 datasets found in our study. 

r-cDWI1000 outperformed r-aDWI1000 with regard to CNR and CR. Furthermore, lesion detection was significantly higher in the r-cDWI1000 datasets than in the directly acquired r-aDWI1000 datasets. Incidence of type 1 tumors was also higher in r-cDWI1000, although it did not reach statistical significance. Overall, the superiority of r-cDWI1000 over r-cDWI1000 is particularly remarkable with the consideration of a scan time reduction of 78% for r-cDWI1000 datasets. However, the rFOV sequence was employed as a study protocol, aiming to ensure an adequate SNR. This was achieved by extensive averaging. rFOV imaging intrinsically suffers from a low SNR, as mentioned previously, and beyond that, the location of the pancreas deep in the abdomen at the center of the FOV further decreases the SNR. In clinical routine, a scan time of 15 min, as performed in our high-resolution, high b-value protocol, would not be feasible. Hence, cDWI enables the acquisition of high b-value datasets in a clinically applicable examination time. 

Our study has some limitations: first, we included only a small patient cohort from a single institution; second, due to the retrospective nature of our study, only measured b-values up to 1000 s/mm^2^ were available (previous studies investigated cDWI for pancreatic cancer at even higher b-values [12,24]); third, we did not investigate other mono-exponential models, such as bi- or tri-exponential diffusion models, which might add further diagnostic value; fourth, we investigated PDAC patients only. Other pancreatic pathologies were not included. Future prospective studies should further elucidate the diagnostic precision of high-resolution cDWI in discriminating between PDAC and other pancreatic pathologies, e.g., mass forming pancreatitis.

## 5. Conclusions

We herein demonstrate the superiority of high-resolution imaging over conventional imaging in computed DWI of PDAC at the high b-value of 1000 s/mm^2^. 

## Figures and Tables

**Table 1:** Displaying patient characteristics.

Category	Variable	*n*
Sex	Male	29 (63%)
	Female	17 (37%)
Age (years)	Mean ± SD	68.9 ± 11.3
Tumor size	cT1	5 (10%)
	cT2	15 (33%)
	cT3	10 (22%)
	cT4	16 (35%)
Nodal status	cN0	24 (52%)
	cN1	12 (26%)
	cN2	10 (22%)
Metastasis	cM0	22 (48%)
	cM1	24 (52%)
CA19-9 (U/mL)	Median	197
	IQR	1025
CEA (ng/mL)	Median	3.61
	IQR	5.88
Tumor location	Head	30 (65%)
	Body	8 (17%)
	Tail	8 (17%)

**Table 2:** Results of *p*-values for comparison of qualitative image parameters. The labeling was corrected.

Category	f-aDWI_600_ vs. f-cDWI_1000_	r-aDWI_600_ vs. r-cDWI_1000_	f-cDWI_1000_ vs. r-cDWI_1000_	r-aDWI_1000_ vs. r-cDWI_1000_
Image quality	<0.00001	0.00222	<0.00001	0.0096
Lesion detection	0.00022	<0.00001	<0.00001	0.041
DWI type	0.277	0.008	0.006	0.41

**Table 3:** Ratings for the assessed qualitative image parameters.

Category	f-aFOV_DWI600_	f-cFOV_DWI1000_	r-aFOV_DWI600_	r-cFOV_DWI1000_	r-aFOV_DWI1000_
Image quality	2.98 ± 0.61	2.15 ± 0.69	3.52 ± 0.62	3.26 ± 0.6	3.5 ± 0.58
Lesion detection	2.34 ± 0.93	2.78 ± 0.88	2.7 ± 0.91	3.67 ± 0.7	3.47 ± 0.68
DWI: type 1	10	15	15	29	23
DWI: type 2	21	20	22	11	17
DWI: type 3	14	7	7	1	2
DWI: type 4	1	4	2	5	4

**Table 4:** *p*-values for comparison of quantitative image parameters.

Sequence	f-aDWI_600_ vs. f-cDWI_1000_	r-aDWI_600_ vs. r-cDWI_1000_	f-cDWI_1000_ vs. r-cDWI_1000_	r-aDWI_1000_ vs. r-cDWI_1000_
aSNR				
*p*-value	0.01778	0.00168	0.00804	0.00438
aCNR				
*p*-value	0.0035	0.00138	0.02088	<0.00001
CR (prox/dist)				
*p*-value	0.00028/0.00014	<0.00001/<0.00001	<0.0401/<0.00001	0.00128/0.0002

## Data Availability

Data are available on request.

## Abbreviations

aCNR: apparent contrast to noise ratio, ADC: apparent diffusion coefficient, aDWI: acquired DWI, aSNR: apparent signal to noise ratio, A/P: anterior/posterior, CA19-9: carbohydrate antigen, CR: contrast ratio, cDWI: computed DWI, CEA: carcinoembryonic antigen, DWI: diffusion-weighted imaging, fFOV: full field-of-view, mpMRI: multiparametric MRI, MRI: magnetic resonance imaging, PDAC: pancreatic ductal adenocarcinoma, rFOV: reduced field-of-view, R/L: right/left, ROI: region of interest, SD: standard deviation, SENSE: sensitivity encoding, SI: signal intensity, ss-EPI: single-shot echo planar imaging, TE: echo time, TR: repetition time, TSE: turbo spin echo, T2w: T2-weighted.

## References

[1] Siegel R.L., Miller K.D., Jemal A. (2019). Cancer statistics, 2019. CA Cancer J. Clin..

[2] Rahib L., Wehner M.R., Matrisian L.M., Nead K.T. (2021). Estimated Projection of US Cancer Incidence and Death to 2040. JAMA Netw. Open.

[3] Singhi A.D., Koay E.J., Chari S.T., Maitra A. (2019). Early Detection of Pancreatic Cancer: Opportunities and Challenges. Gastroenterology.

[4] Toft J., Hadden W.J., Laurence J.M., Lam V., Yuen L., Janssen A., Pleass H. (2017). Imaging modalities in the diagnosis of pancreatic adenocarcinoma: A systematic review and meta-analysis of sensitivity, specificity and diagnostic accuracy. Eur. J. Radiol..

[5] Raman S.P., Horton K.M., Fishman E.K. (2012). Multimodality imaging of pancreatic cancer-computed tomography, magnetic resonance imaging, and positron emission tomography. Cancer J..

[6] Messina C., Bignone R., Bruno A., Bruno A., Bruno F., Calandri M., Caruso D., Coppolino P., Robertis R.D., Gentili F. (2020). Diffusion-Weighted Imaging in Oncology: An Update. Cancers.

[7] Bammer R. (2003). Basic principles of diffusion-weighted imaging. Eur. J. Radiol..

[8] Barral M., Taouli B., Guiu B., Koh D.M., Luciani A., Manfredi R., Vilgrain V., Hoeffel C., Kanematsu M., Soyer P. (2015). Diffusion-weighted MR imaging of the pancreas: Current status and recommendations. Radiology.

[9] Kaissis G., Ziegelmayer S., Lohöfer F., Algül H., Eiber M., Weichert W., Schmid R., Friess H., Rummeny E., Ankerst D. (2019). A machine learning model for the prediction of survival and tumor subtype in pancreatic ductal adenocarcinoma from preoperative diffusion-weighted imaging. Eur. Radiol. Exp..

[10] Fukukura Y., Takumi K., Kamimura K., Shindo T., Kumagae Y., Tateyama A., Nakajo M. (2012). Pancreatic adenocarcinoma: Variability of diffusion-weighted MR imaging findings. Radiology.

[11] Crinò S.F., Larghi A., Bernardoni L., Parisi A., Frulloni L., Gabbrielli A., Parcesepe P., Scarpa A., Manfrin E. (2019). Touch imprint cytology on endoscopic ultrasound fine-needle biopsy provides comparable sample quality and diagnostic yield to standard endoscopic ultrasound fine-needle aspiration specimens in the evaluation of solid pancreatic lesions. Cytopathology.

[12] Fukukura Y., Kumagae Y., Hakamada H., Shindo T., Takumi K., Kamimura K., Nakajo M., Umanodan A., Yoshiura T. (2017). Computed diffusion-weighted MR imaging for visualization of pancreatic adenocarcinoma: Comparison with acquired diffusion-weighted imaging. Eur. J. Radiol..

[13] Ichikawa S., Kromrey M.L., Motosugi U., Onishi H. (2021). Optimal target b-value on computed diffusion-weighted magnetic resonance imaging for visualization of pancreatic ductal adenocarcinoma and focal autoimmune pancreatitis. Abdom. Radiol..

[14] Dietrich O., Biffar A., Baur-Melnyk A., Reiser M.F. (2010). Technical aspects of MR diffusion imaging of the body. Eur. J. Radiol..

[15] Akagi M., Nakamura Y., Higaki T., Matsubara Y., Terada H., Honda Y., Tatsugami F., Baba Y., Iida M., Awai K. (2018). Preliminary Results of High-Precision Computed Diffusion Weighted Imaging for the Diagnosis of Hepatocellular Carcinoma at 3 Tesla. J. Comput. Assist. Tomogr..

[16] Rosenkrantz A.B., Chandarana H., Hindman N., Deng F.M., Babb J.S., Taneja S.S., Geppert C. (2013). Computed diffusion-weighted imaging of the prostate at 3 T: Impact on image quality and tumour detection. Eur. Radiol..

[17] Tamura T., Takasu M., Higaki T., Yokomachi K., Akiyama Y., Sumida H., Nagata Y., Awai K. (2019). How to Improve the Conspicuity of Breast Tumors on Computed High b-value Diffusion-weighted Imaging. Magn. Reson. Med. Sci. MRMS Off. J. Jpn. Soc. Magn. Reson. Med..

[18] Gatidis S., Schmidt H., Martirosian P., Nikolaou K., Schwenzer N.F. (2016). Apparent diffusion coefficient-dependent voxelwise computed diffusion-weighted imaging: An approach for improving SNR and reducing T2 shine-through effects. J. Magn. Reson. Imaging JMRI.

[19] Harder F.N., Kamal O., Kaissis G.A., Heid I., Lohöfer F.K., McTavish S., Van A.T., Katemann C., Peeters J.M., Karampinos D.C. (2020). Qualitative and Quantitative Comparison of Respiratory Triggered Reduced Field-of-View (FOV) Versus Full FOV Diffusion Weighted Imaging (DWI) in Pancreatic Pathologies. Acad. Radiol..

[20] Ma C., Li Y.J., Pan C.S., Wang H., Wang J., Chen S.Y., Lu J.P. (2014). High resolution diffusion weighted magnetic resonance imaging of the pancreas using reduced field of view single-shot echo-planar imaging at 3 T. Magn. Reson. Imaging.

[21] Cho E., Lee J.H., Baek H.J., Ha J.Y., Ryu K.H., Park S.E., Moon J.I., Gho S.M., Wakayama T. (2020). Clinical Feasibility of Reduced Field-of-View Diffusion-Weighted Magnetic Resonance Imaging with Computed Diffusion-Weighted Imaging Technique in Breast Cancer Patients. Diagnostics.

[22] Ning P., Shi D., Sonn G.A., Vasanawala S.S., Loening A.M., Ghanouni P., Obara P., Shin L.K., Fan R.E., Hargreaves B.A. (2018). The impact of computed high b-value images on the diagnostic accuracy of DWI for prostate cancer: A receiver operating characteristics analysis. Sci. Rep..

[23] Fukukura Y., Shindo T., Hakamada H., Takumi K., Umanodan T., Nakajo M., Kamimura K., Umanodan A., Ideue J., Yoshiura T. (2016). Diffusion-weighted MR imaging of the pancreas: Optimizing b-value for visualization of pancreatic adenocarcinoma. Eur. Radiol..

[24] Tokunaga K., Arizono S., Shimizu H., Fujimoto K., Kurata M., Minamiguchi S., Isoda H., Togashi K. (2020). Optimizing b-values for accurate depiction of pancreatic cancer with tumor-associated pancreatitis on computed diffusion-weighted imaging. Clin. Imaging.

[25] Higaki T., Nakamura Y., Tatsugami F., Kaichi Y., Akagi M., Akiyama Y., Baba Y., Iida M., Awai K. (2018). Introduction to the Technical Aspects of Computed Diffusion-weighted Imaging for Radiologists. Radiogr. Rev. Publ. Radiol. Soc. N. Am. Inc..

[26] Bittencourt L.K., Attenberger U.I., Lima D., Strecker R., de Oliveira A., Schoenberg S.O., Gasparetto E.L., Hausmann D. (2014). Feasibility study of computed vs measured high b-value (1400 s/mm²) diffusion-weighted MR images of the prostate. World J. Radiol..

[27] Grant K.B., Agarwal H.K., Shih J.H., Bernardo M., Pang Y., Daar D., Merino M.J., Wood B.J., Pinto P.A., Choyke P.L. (2015). Comparison of calculated and acquired high b value diffusion-weighted imaging in prostate cancer. Abdom. Imaging.

[28] Saritas E.U., Cunningham C.H., Lee J.H., Han E.T., Nishimura D.G. (2008). DWI of the spinal cord with reduced FOV single-shot EPI. Magn. Reson. Med..

[29] Wilm B.J., Svensson J., Henning A., Pruessmann K.P., Boesiger P., Kollias S.S. (2007). Reduced field-of-view MRI using outer volume suppression for spinal cord diffusion imaging. Magn. Reson. Med..

[30] Zhang Y., Wells S.A., Triche B.L., Kelcz F., Hernando D. (2020). Stimulated-echo diffusion-weighted imaging with moderate b values for the detection of prostate cancer. Eur. Radiol..

[31] Wargo C.J., Moore J., Gore J.C. (2013). A comparison and evaluation of reduced-FOV methods for multi-slice 7T human imaging. Magn. Reson. Imaging.

[32] Blackledge M.D., Leach M.O., Collins D.J., Koh D.M. (2011). Computed diffusion-weighted MR imaging may improve tumor detection. Radiology.

